# Titania (TiO_2_) nanoparticles enhance the performance of growth-promoting rhizobacteria

**DOI:** 10.1038/s41598-017-18939-x

**Published:** 2018-01-12

**Authors:** Salme Timmusk, Gulaim Seisenbaeva, Lawrence Behers

**Affiliations:** 1 Department of Forest Mycology and Plant Pathology, Uppsala BioCenter, SLU, SE-75007 Uppsala, Sweden; 2 Department of Molecular Sciences, Uppsala BioCenter, SLU, SE-75007 Uppsala, Sweden; 3 The Bashan Institute of Science 1730 Post Oak Court, Auburn, AL 36830 USA

## Abstract

A novel use of nanotitania (TNs) as agents in the nanointerface interaction between plants and colonization of growth promoting rhizobacteria (PGPR) is presented. The effectiveness of PGPRs is related to the effectiveness of the technology used for their formulation. TNs produced by the Captigel patented SolGel approach, characterized by the transmission and scanning electron microscopy were used for formulation of the harsh environment PGPR strains. Changes in the biomass of wheat seedlings and in the density of single and double inoculants with and without TNs were monitored during two weeks of stress induced by drought salt and by the pathogen *Fusarium culmorum*. We show that double inoculants with TNs can attach stably to plant roots. Regression analysis indicates that there is a positive interaction between seedling biomass and TN-treated second inoculant colonization. We conclude that TN treatment provides an effectual platform for PGPR rational application via design of root microbial community. Our studies illustrate the importance of considering natural soil nanoparticles for PGPR application and thereby may explain the generally observed inconsistent behavior of PGPRs in the field. These new advancements importantly contribute towards solving food security issues in changing climates. The model systems established here provide a basis for new PGPR nanomaterials research.

## Introduction

Agriculture faces several challenges at a global level, owing to climate change and resource limitation. Crop production is limited due to various abiotic stresses such as drought, salt and pollutants, as well as frequent infections of plant pathogens and nutritional limitations. In the face of these challenges, crop yield must be maintained and improved using fewer inputs. Such an approach will require complex strategies combining environmental and agricultural practices with innovative approaches, to successfully counteract the negative effects of climatic stress. Soil is a ubiquitous habitat with great taxonomic and functional diversity of microorganisms^1–5^. The bacteria are known to help plant growth by a combination of physiological attributes, such as non-symbiotic N_2_ fixation, phytohormone production, solubilization of insoluble mineral phosphate and siderophore production^3,6,7^. Plant growth promoting rhizobacteria **(**PGPR) protect plants against biotic and abiotic stress situations and contribute to restoring marginal lands via enhancing soil biodiversity and phytoremediation^3,7–16^. PGPR applications are performed as single inoculations but also in the more complex form as multiple inoculations. The complex inoculations, mostly dual inoculations, may have several advantages over single inoculation^17^. Development of methods for the robust and efficient application of PGPRs in natural environments, where multiple factors act simultaneously, has been attempted by many researchers over a long period of time. It is clear that detailed approaches of how PGPRs interact with the host and surrounding environments has to be focused in order to ensure their wide scale effective and reproducible application in natural environments^18–20^. Usually bacteria are added as suspensions to seed surfaces, root surfaces or into soil. Colonization by the bacterium is influenced by the bacterial properties as well as the physical, chemical and biological nature of the environment. Various factors affect the bacterial colonization, e.g. soil particle aggregation, quantity and quality of available C, temperature, and pH to name a few. Usually the supplied bacterial population declines rapidly, making it difficult to maintain activity in the rhizosphere. Consequently, the PGPRs have to be prepared methodically, in order to provide an appropriate micro-environment combined with physical protection for a sustained period of time to avoid decline^18,21^. PGPR preparation, called formulation, makes it possible to deliver a reliable source of bacteria that survives and becomes available to crops, when necessary. Usually PGPR cells are entrapped in some gel matrix substrates whose products are easily circulated, e.g. self-produced biofilm matrix, agar, agarose, collagen or alginate^21–24^.

The solid phase of soil is composed of minerals (inorganic) and organic material. Minerals predominate in virtually all soils. The most abundant class of minerals are silicates, which have a substantial impact on soil characteristics, as their surfaces are inherently reactive, potentially forming strong or weak chemical bonds with soluble substances and further regulating the composition of the soil solution^25^. The plant’s ability to produce exudates that can dissolve the surrounding mineral background is a well-known property of plant metabolism. This property is indispensable for adaptation in stress situations. It has also become clear that the role of root exudates is complex and includes plants’ communication with surrounding soil microorganisms^26^. Recent research revealed that release of chelating organic acids and their anions from the plant roots not only dissolves minerals, but provide a mechanism for generating mineral nanoparticles (NPs) that are fully biocompatible with plants^27–29^. An extremely large surface area of NPs results in strong adsorption of various molecules that reduce nanoparticle surface energy^25^. Thus, pristine NPs are nearly nonexistent. Interactions between NPs and biological systems (*viz*. nano−bio interactions) are driven by drastic modifications of NPs in biological media, such as formation of NP complexes, which are in turn strongly dependent on the chemical properties of the NPs^25^. It is clear that in order for the PGPRs to be used most effectively, there needs to be a rational approach in providing a formulation and delivery of specific PGPRs or their bioactive products in the field. As suggested earlier, the development of mathematical models-based “customized” inocula would facilitate the stable and reproducible application of PGPRs^18^. Compared to other formulation technologies NPs stand out, as they can be employed to deliver PGPRs and their active chemical agents in a regulated manner, i.e. focusing on specific types of cells or tissues, at specific times. Although NPs are found in all natural environments, they have to be produced for biotechnological purposes. Titania (TiO_2_) nanoparticles (TNs) have become an integral part of nanotechnology in several fields^30,31^. Small TNs can be produced in a fully biocompatible manner using sol-gel technology^30,31^ and have been demonstrated to be environmentally benign using tobacco pollen growth as an indicator^29^.

Rhizobacterial ability to enhance plant drought stress adaptation was discovered in 1999 by Timmusk and Wagner, in an attempt to study the soil bacterium *P*. *polymyxa* B2 strain for plant-induced resistance and nitrogen fixation ability^32^. Since then several *P*. *polymyxa* and *Bacillus thuringiensis* strains from contrasting environments have been isolated, and it has been shown that the bacteria from harsh environments are more likely to be efficient in enhancing host plant stress tolerance^4,9,33–35^. We have shown that *P*. *polymyxa A26* and *B*. *thuringiensis AZP2* can enhance plant drought stress tolerance and that strain A26 has biocontrol activity against *Fusarium culmorum and F*. *graminearum* (Table 1). The *P*. *polymyxa* A26 Sfp-type 4-phosphopantetheinyl transferase deletion mutant strain (A26∆sfp), lacking the ability to produce both nonribosomal peptides and polyketides, was created and we showed that the Sfp-type PPTase gene in *P*. *polymyxa* is a gate-keeper for bacterial drought tolerance enhancement^9,34,36^. The harsh environmental isolates, as well as the mutant strain were used in this study.

**Table 1:** Strains used in the study.

**Name**	**Abbreviation**	**Origin**	**Publications**
*Bacillus thuringiensis AZP2*	AZP2	Ponderosa pine rhizosphere, Mt. Lemmon, AZ, USA	Timmusk *et al*., 2014b
*Paenibacillus polymyxa* A26	A26	Wild barley rhizosphere, Evolution Canyon, Haifa, Israel	Timmusk *et al*., 2011, Timmusk *et al*., 2014b, Abd El-Daim *et al*., 2015
*Paenibacillus polymyxa* A26∆sfp	A26∆sfp	Wild barley rhizosphere, Evolution Canyon, Haifa, Israel	Kim &Timmusk, 2013 Timmusk *et al*. 2014 Abd El-Daim *et al*. 2015
*Alcaligenes faecalis* AF	AF	Ponderosa pine rhizosphere, Mt. Lemmon, AZ, USA	This study
*Fusarium culmorum UK*	FcUK	Wheat rhizosphere, UK	Pasquali *et al*., 2013

The general aim of our work is to develop a quantitative base for interacting functional information from environment and microbial networks to whole plant phenotypic performance. In the current study we examined how nanoparticles affect the PGPR performance under drought, salt and pathogen stress. We show that PGPRs with TNs form stable robust microbial layers and enhance the positive effect of double inoculation. To the best of our knowledge this is the first report to show that biocompatible titania nanoparticles aid performance of the bacteria under stress and can be effectively used for PGPR formulation in the case of several inoculants. The results draw attention to the need to consider existing soil nanoparticles when applying the TN formulation technology.

## Results

### Characteristics of the nanoparticles

**Figure 1:**
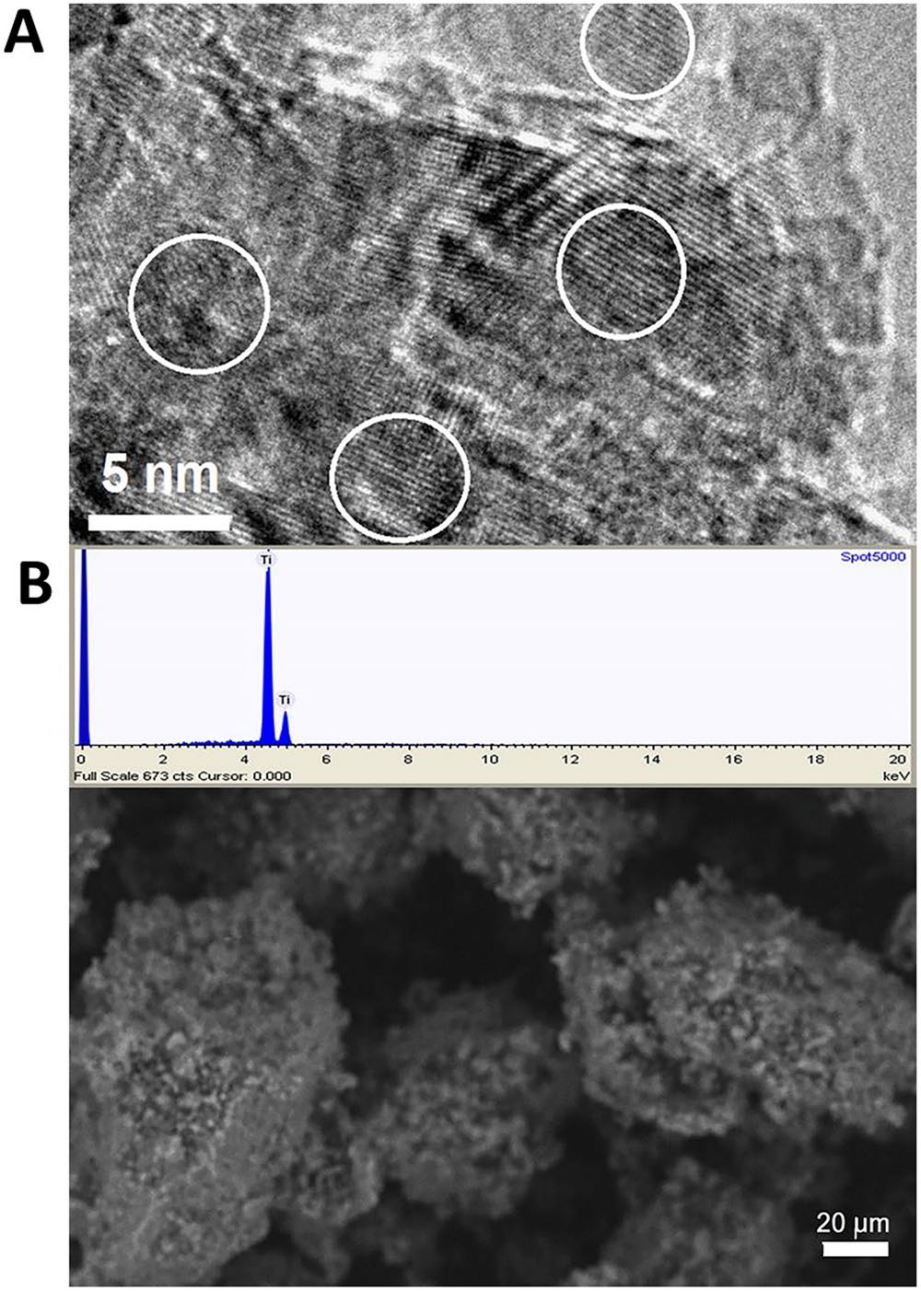
Titania nanoparticles used in the study. Transmission electron microscopy (**A**) environmental scanning electron microscopy micrographs and energy dispersive X-ray spectroscopy (**B**) of the SolGel-produced TN particles.

**Figure 2:**
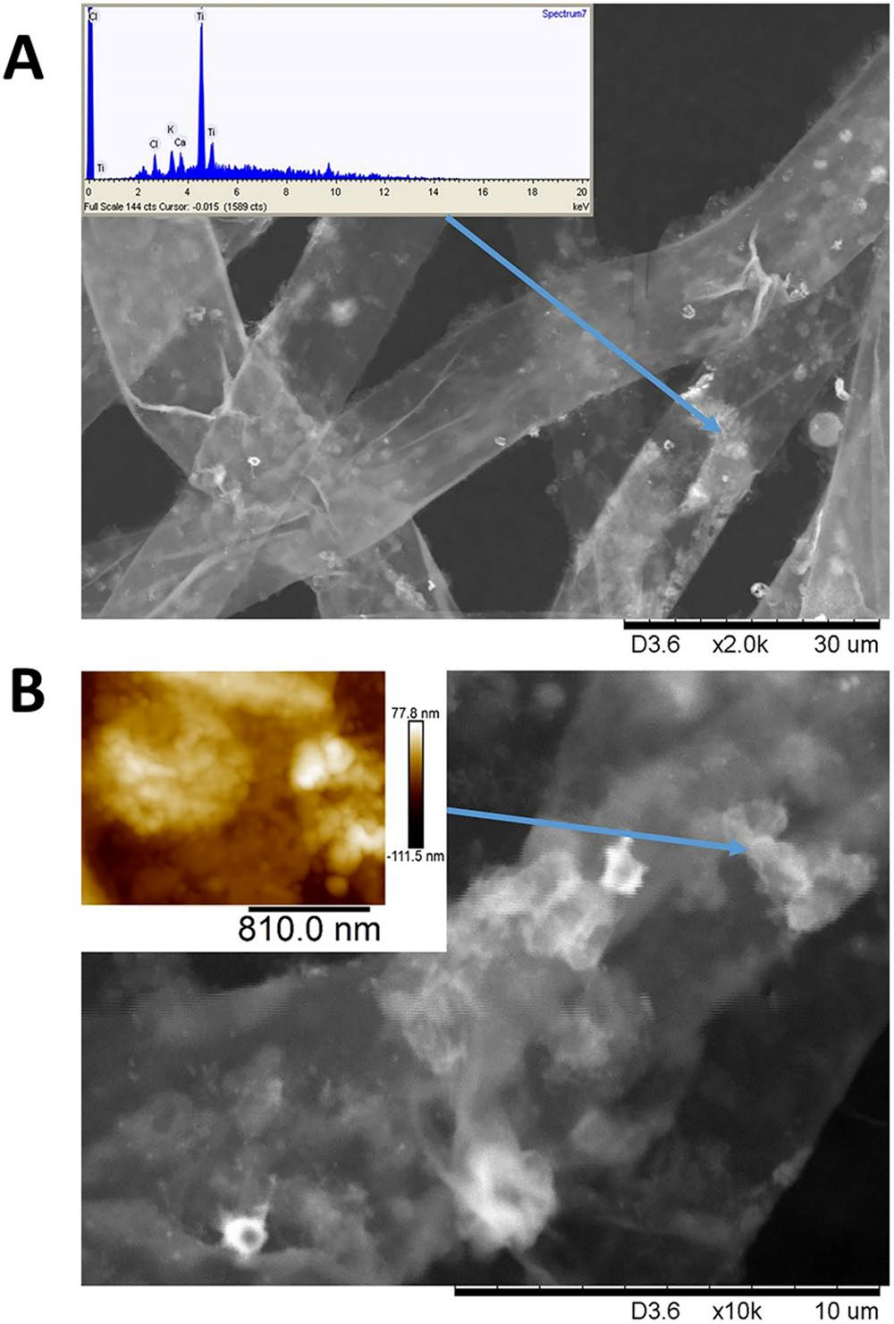
PGPR TN aggregates on plant root. Typical ESEM-EDS images of PGPR cells grown with TNs for 24 hours on plant root after 6 hours of inoculation (**A**). and the characteristic aggregate sizes of 50–60 nm (**B**).

The TNs were studied by TEM and ESEM and found to exist as relatively uniform 5 nm particles in aggregates (Fig. 1A and B). The fluffy agglomerated solid of the TNs was easily peptized and de-agglomerated by ultrasound treatment over 1 to 5 min. The ultrasound treated colloid was pH-neutral (7–7.5). When the TNs were supplied to the PGPR growth medium, dense and coarse bacterial clumps were formed which were more developed than the clumps formed in the case of self-produced biofilm (Fig. S2 A and B). The aggregates of TNs were clearly visible on the surface of bacteria in both SEM and AFM images (where the characteristic sizes of 50–60 nm could clearly be traced, (Fig. 2). The area EDS analysis of the biofilms was showing 7.5% of Ti in average among the heavier elements, while the spot analysis on nanoparticle aggregates revealed 70–85% of Ti, showing that the particles were present in undigested form.

**Figure 3:**
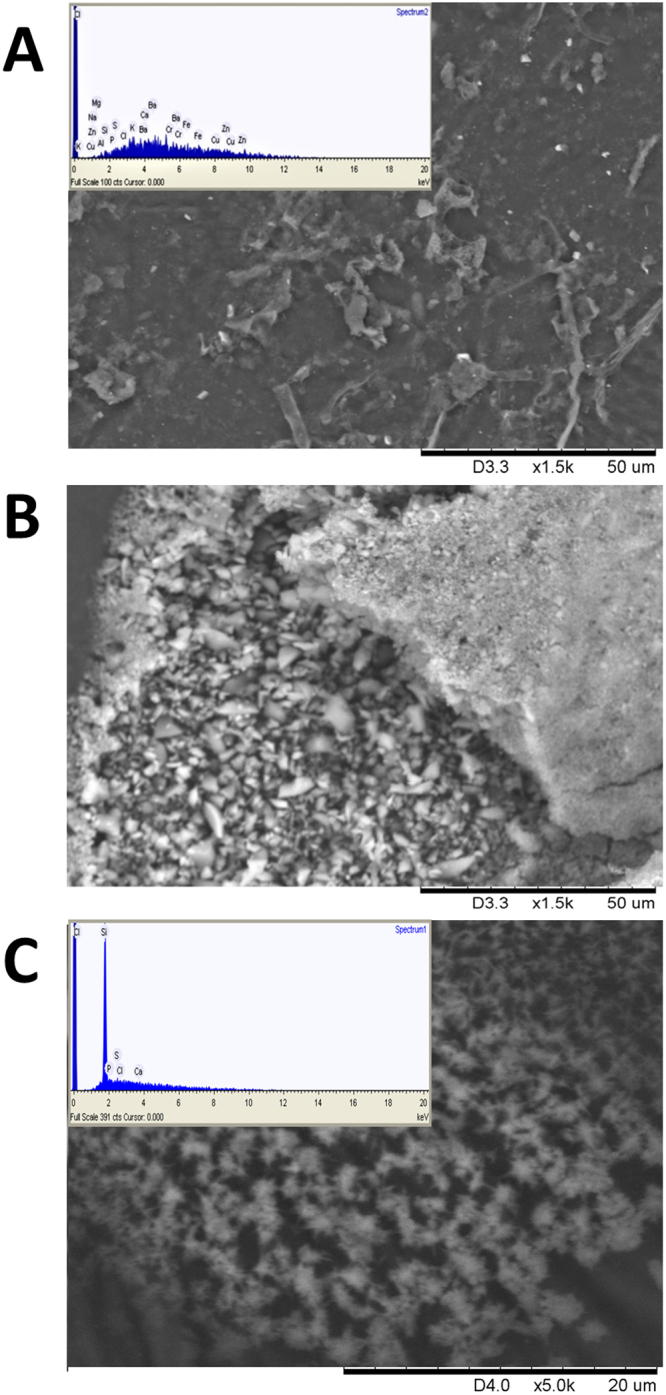
Peat soil and sand used in the study. ESEM micrographs of peat soil (**A**) sand (**B**) and 0.2 µm filtrates of sand (**C**). The ESEM-EDS analysis of the filtrates showed that peat contains traces of various elements (**A**) but no nanoparticles. Sand used in the study is abundant in Si nanoparticles (**C**).

**Figure 4:**
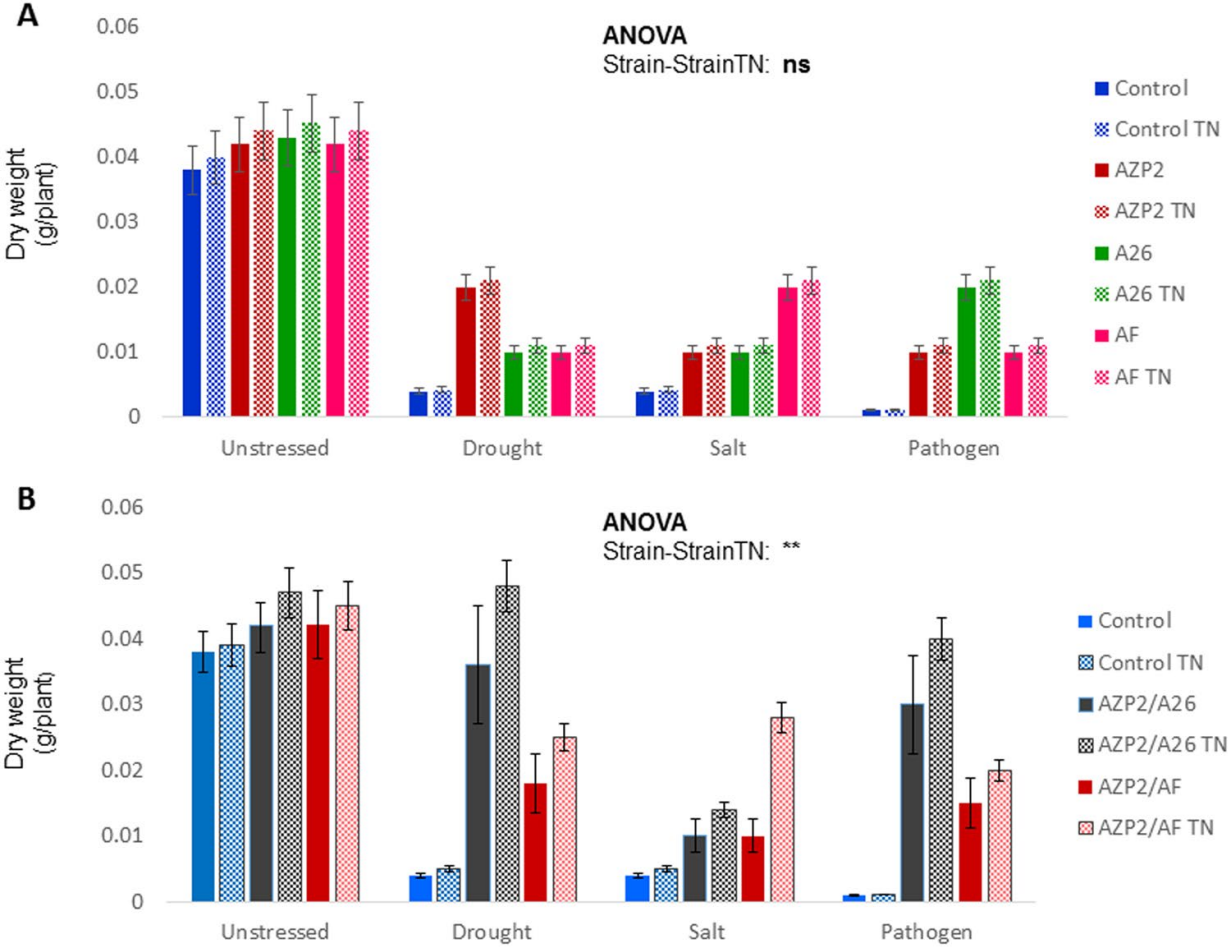
PGPR inoculation with and without TNs in peat soil. (**A**) Single inoculation. *Bacillus thuringiensis* AZP2, *Paenibacillus polymyxa* A26 and *Alcaligenes faecalis* AF impact on seedlings dry weight under drought, salt and pathogen (*Fusarium culmorum* UK) stress. The error bars indicate ± SE for three biological replicates. Statistical analysis is based on ANOVA with strains (AZP2, A26 and AF) TN formulation (TN^+^ and TN^−^) as factors. ** indicates significant (*p* < 0.01) and ns non-significant effect of TN treatment. (**B**) Double inoculation. AZP2/A26 and AZP2/AF effect on seedlings dry weight under the three stress situations (drought, salt and pathogen *F*. *culmorum* UK). The error bars indicate ± SE for five biological replicates. Statistical analysis of significance as in Fig. 4A.

**Figure 5:**
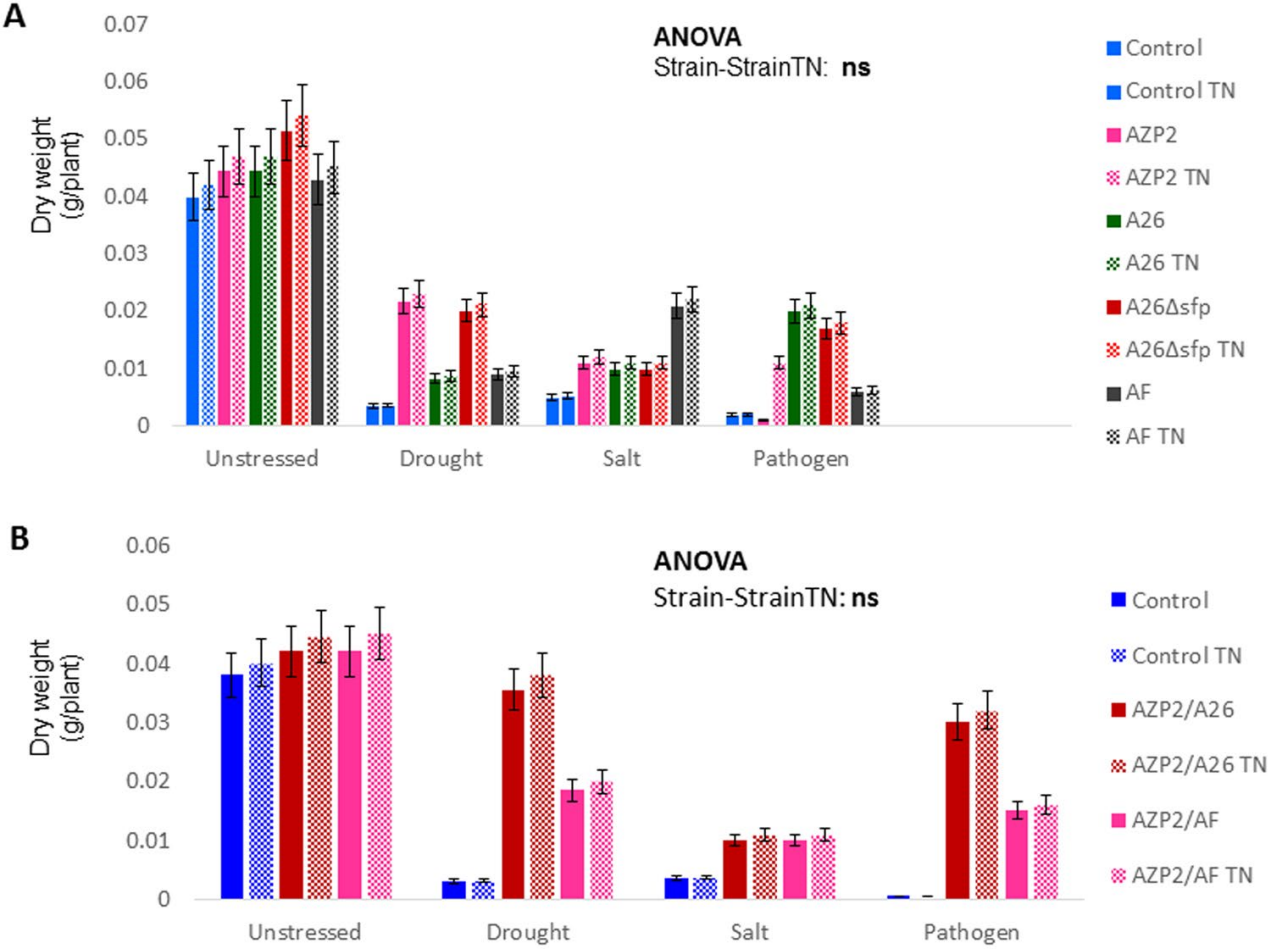
PGPR inoculation with and without TNs in sand. (**A**) Single inoculation. *Bacillus thuringiensis* AZP2, *Paenibacillus polymyxa* A26 and *Alcaligenes faecalis* AF impact on seedlings dry weight under drought, salt and pathogen (*Fusarium culmorum* UK) stress. The error bars indicate ± SE for three biological replicates. Statistical analysis is based on ANOVA with strains (AZP2, A26, A26sfp and AF) TN formulation (TN^+^ and TN^−^) as factors. ** and ns, indicate significant (*p* < 0.01) or non-significant effect of TN treatment. (**B**) Double inoculation. AZP2/A26 and AZP2/AF effect on seedlings dry weight under the three stress situations (drought, salt and pathogen *F*. *culmorum* UK). The error bars indicate ± SE for five biological replicates. Statistical analysis of significance as in Fig. 4A.

The experiments were performed in two growth substrates of contrasting Si nanoparticle abundance. The SEM-EDS analysis of the substrate 0.2 µm filtrates showed that peat contains traces of various elements but no nanoparticles (Fig. 3). Sand used in the study is abundant in Si nanoparticles (Fig. 3). The biomass accumulation of the wheat cv Stava was used to assess the effect of PGPRs with and without TNs in peat (Fig. 4) and sand (Fig. 5) under three stress situations (drought, salt and pathogen stress).

### PGPR inoculation with and without TNs in peat soil

**Figure 6:**
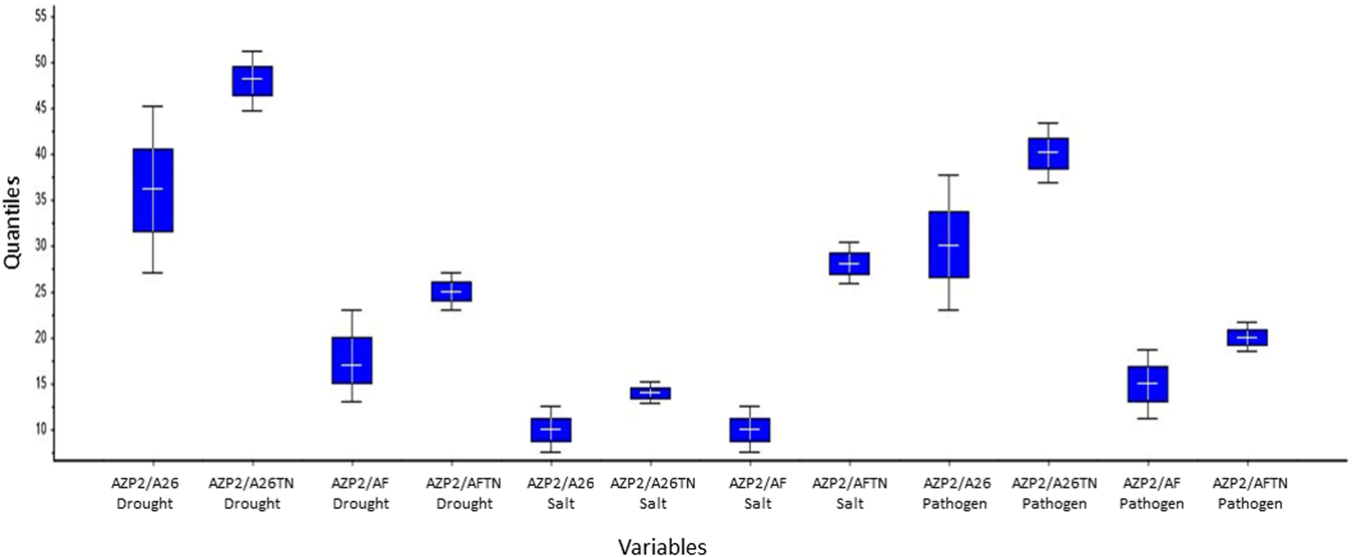
Plant biomass distribution of seedlings inoculated with PGPR (with and without TNs). The box plot shows minimum, first quartile, median, third quartile and maximum of the AZP2, A26 and AF combinations under drought, salt and pathogen stress.

The single inoculations with AZP2, A26, AF and double inoculations with AZP2/A26 AZP2/AF slightly improved biomass under unstressed conditions (Fig. 4A,B, Table S1). In contrast to the slight improvement under unstressed condition, more than five-fold biomass improvement was observed by AZP2 inoculation under drought stress (*p* < 0.01), by AF under salt stress (*p* < 0.01), and by A26 under pathogen stress (*p* < 0.01). Inoculation with A26 and AF under drought, AZP2 and A26 under salt and AZP2 and AF under pathogen stress, resulted in lower but significant biomass increase (two to three times) (*p* < 0.01) (Fig. 4A). Double inoculations, i.e AZP2 followed by A26 and AF, resulted in significant plant dry weight increases (Table S1). However, the improvement in biomass varied greatly (Figs 4B and 6). TNs did not improve seedling biomass when single strain inoculations were used (Fig. 4A). Fascinatingly, major differences in seedlings biomass were observed when double inoculations with TNs were performed in peat soil. AZP2TN and A26TN as well as AZP2TN and AFTN combinations resulted in a stable, about a 25% plant biomass increase (Figs 4B, 6, Table S1).

**Figure 7:**
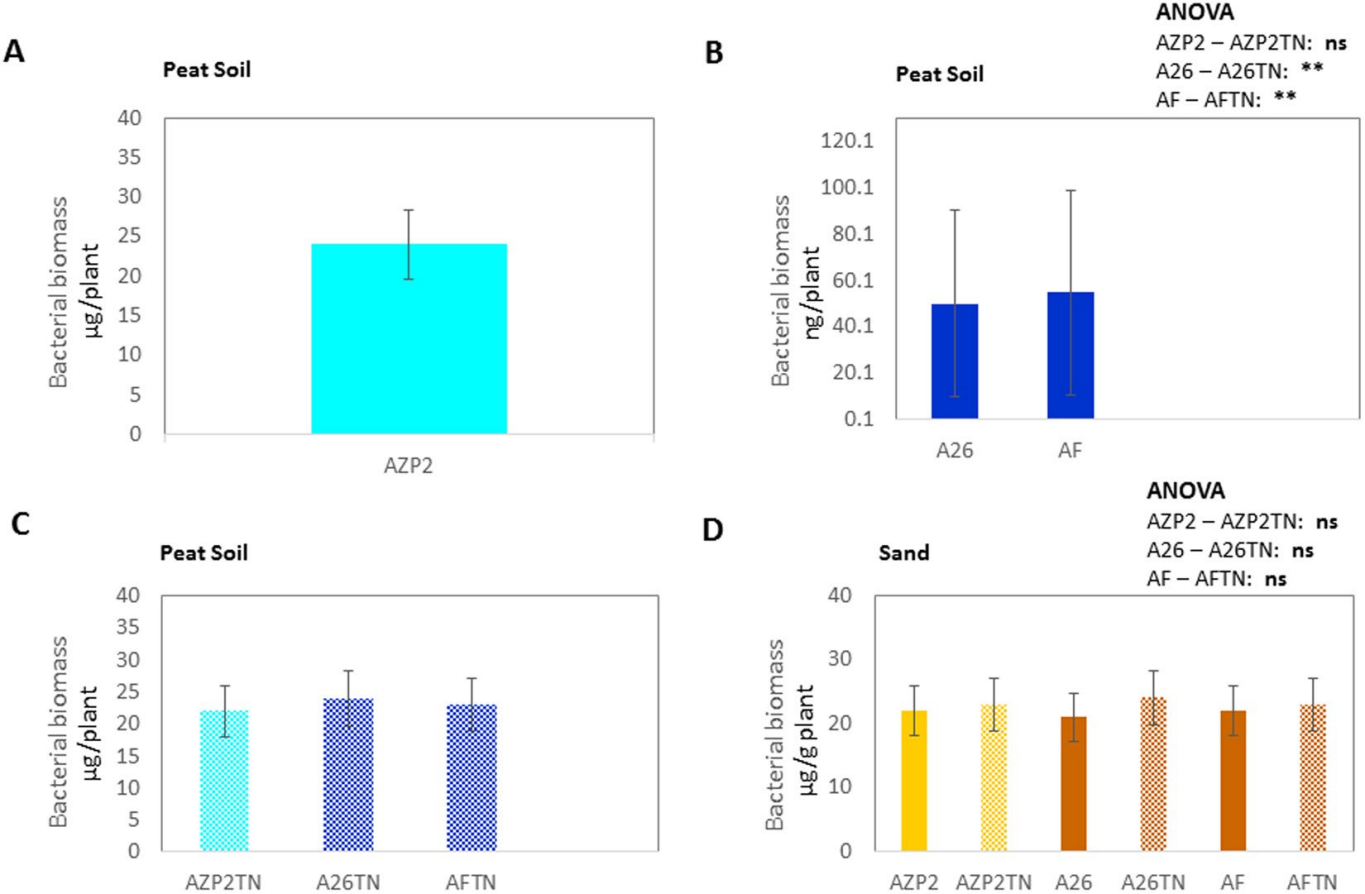
TN treatment effect on bacterial colonization (bacterial biomass). (**A)** First inoculant AZP2 (light blue) in peat soil; (**B**)Second inoculants A26 and AF (dark blue) in peat soil, (**C**) First and second inoculants with TNs A26TN and AFTN in peat soil, (**D**) First and second inoculants in sand. Given as means with 95% confidence intervals. **p < 0.001; ns nonsignificant. Bacterial biomass (C-content) calculated as described by Bratbak, 1985 was used as a proxy for root colonization plant biomass regression analysis.

**Figure 8:**
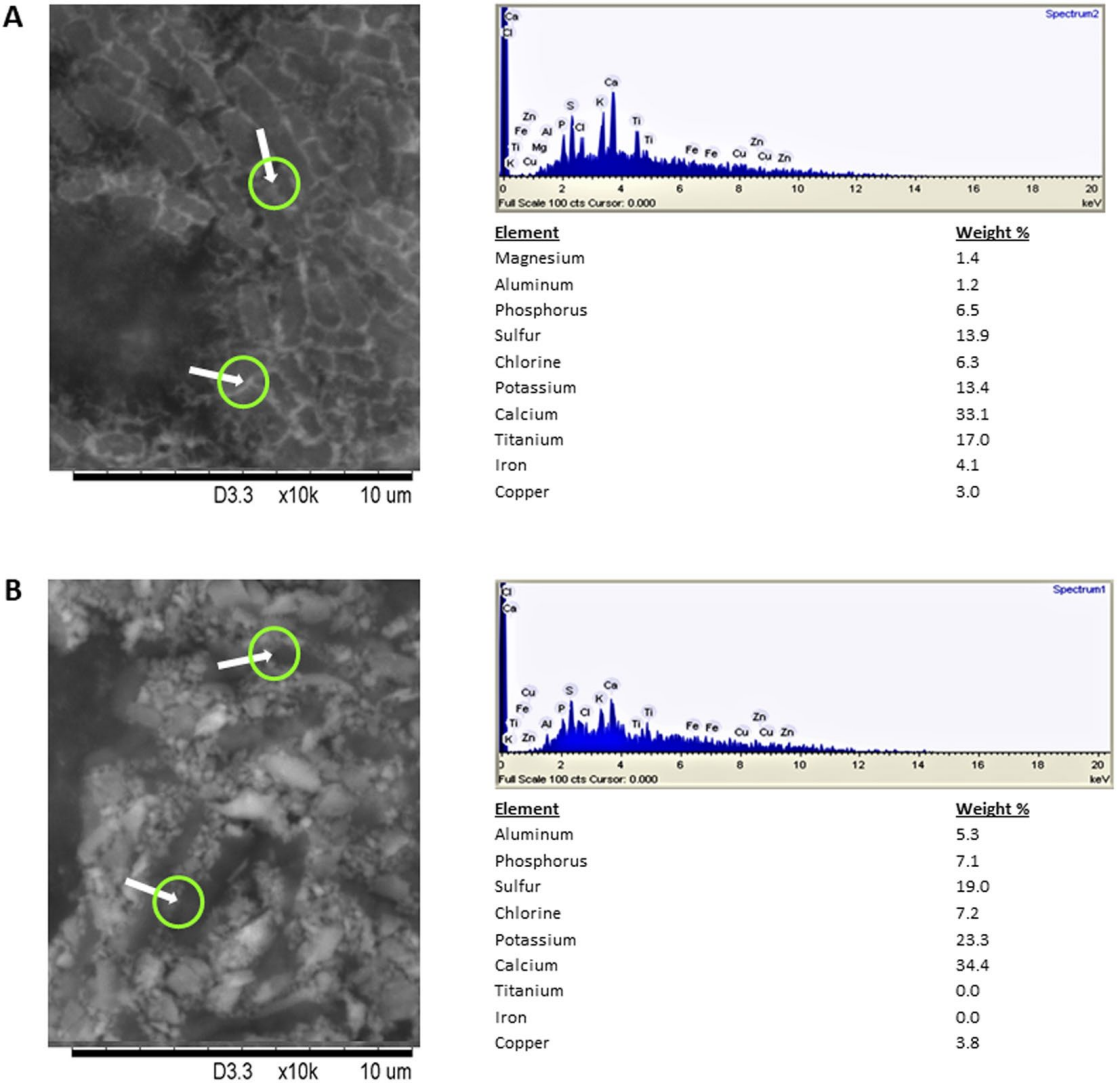
TN treatment effect on second inoculant rhizosphere colonization. Typical ESEM image and energy dispersive X-ray spectroscopy results of TN treated second inoculant colonization (**A**) and second inoculant colonization without TNs (**B**) in peat soil after two days of inoculation. Arrows indicate bacterial cells.

**Figure 9:**
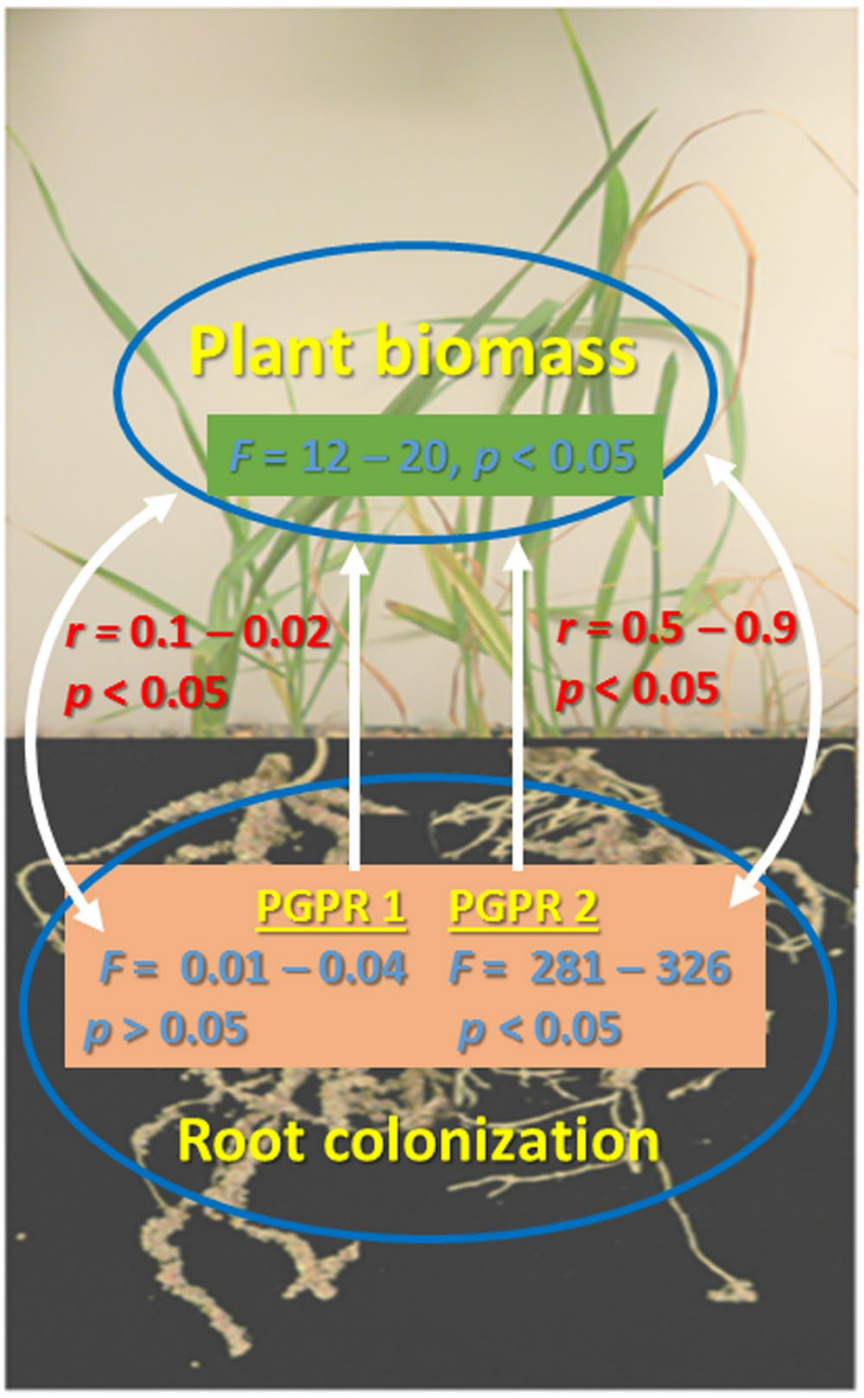
Relationships between plant biomass accumulation and PGPR colonization related to TN treatment. PGPR TN treatment effect on colonization and wheat seedlings biomass (blue). Correlations among PGPR1 and PGPR2 colonization and plant biomass accumulation (red). One headed arrows indicate treatment effects while double headed arrows indicate correlations. Note the significant effect of TN treatment on PGPR2 colonization. See Table S1, Table S2 and Table S3 for details.

The fate of the three strains (AZP2, A26 and AF) and their combinations (AZP2/A26 AZP2/AF) with and without TNs was followed using selection plates and by PCR. 10^4^-10^5^ bacterial cfu per gram of plant material after 24 days of plant growth was detected in the case of single inoculations. In the case of double inoculation 10^4^-10^5^ cfu of the first inoculant AZP2 was always detected per gram of plant material (Fig. 7A). Without TNs considerable fluctuation occurred in the detection results of the second inoculant (A26 or AF) and sometimes just traces were detected (Fig. 7B). When TN formulation was used, 10^4^-10^5^cfu of the first as well as second inoculant were detected per gram of plant material (Fig. 7C). Hence, AZP2TN and A26TN as well as AZP2TN and AFTN combinations resulted in significantly enhanced colonization (Figs 7C and 8A). ESEM-EDS and ANOVA results confirm highly significant colonization effect due the second inoculant A26 or AF TN treatment under drought (*F*-ratio 290, 326, *p* < 0.001 respectively), salt (*F*-ratio 281, 299, *p* < 0.001 respectively) and pathogen stress (*F*-ratio 290, 292, *p* < 0.001 respectively) (Table S2, Figs 7, 8 and 9). Regression analysis indicates that there is a positive interaction between TN-formulated second PGPR and seedling biomass. Plant biomass increases with increased colonization of the second inoculant A26 or AF under drought (*r* = 0.59, 0.61; *p* = 0.06, 0.35 respectively), salt (*r* = 0.54, 0.89; *p* = 0.014, 0.01 respectively) and pathogen stress (*r* = 0.47, 0.46; *p* = 0.035, 0.04 respectively) (Table S3 and Fig. 9).

### PGPR inoculation with and without TNs in sand

Similar to the peat soil studies, the effect of the harsh environment PGPRs with and without TNs under three stress situations (drought, salt and pathogen stress) were analyzed. In contrast to the slight improvement under unstressed condition, more than five-fold biomass improvement was observed by AZP2 inoculation under drought, by AF under salt, and by A26 under pathogen stress (Fig. 5A). The improvement was highly significant at a conservative level of significance of *p* ≤ 0.01. A26, AZP2 and AF inoculations resulted in lower but significant dry weight increases under drought, salt and pathogen stress, respectively. Figure 5B illustrates that AZP2 priming followed by A26 and AF priming improved the AZP2 plant growth promoting effect. Wheat dry weight improved two-fold in combination with A26 under drought stress and pathogen stress (Fig. 5B). AF enhanced the AZP2 growth-promoting effect 2.2 and 1.6 times under salt and pathogen stress (Fig. 4B). Neither the single nor double PGPR TN formulations resulted in significant differences at the *p* value ≤ 0.01.

The fate of the PGPRs and their combinations was followed and after 24 days of plant growth the seedling root-associated bacteria were quantified. 10^4^-10^5^ cfu of the first and second inoculants were detected per gram of plant material. ANOVA results show that in contrast to peat soil there is no significant effect of the second inoculant (A26 or AF) TN treatment to their root colonization under drought, salt nor pathogen stress (Fig. 7D).

## Discussion

The TNs in this work were obtained by sol-gel approach, exploiting the technique, modified from the earlier reported by us in^31^ to facilitate the separation of the material. The TNs were shown to be fully biocompatible and did not produce any negative bio-effects on sensitive plant models such as tobacco pollen even with concentrations as high as 100 µg ml^−1^ ^31^. TNs have the ability to be biodegraded in the presence of chelating carboxylate ligands such as citrate, oxalate and lactate, which are excreted by plant root systems. This property may explain the titania conversion to naturally abundant soil minerals^27^.

The hydrolytic treatment of metal alkoxides is well demonstrated to produce rather small (3–5 nm) uniform particles^37^. It is important to note the difference in appearance and behavior of such particles compared to broadly used Degussa P25 or colloidal titania of US Research Nanomaterials (Product No US 7070) that are often utilized as standard in biological experiments (see, for example,^38^). The latter particles are relatively large, 30–50 nm, polyhedron-shaped single crystals. Sol-gel TNs are approximately 10 times smaller and possess much larger surface area and much higher related chemical reactivity. In solution in mother liquor or MilliQ water they remain de-aggregated as can be visualized by DLS, but in biological media easily form aggregates with the size of about 50 nm and bigger (see Supplementary, FS1) that, however can easily be split by sonication to almost the primary size. The structure of such aggregate revealed by high-resolution TEM (Fig. 1A) features individual particles randomly set together. The analysis of distances between the adjacent fringes (0.35 nm) indicated the anatase phase. The EDS analysis features high purity titania (Fig. 1B), while SEM features aggregates of fluffy material compacted on drying. The prepared TNs were easily peptized and de-agglomerated by ultrasound treatment in 1–5 min. The washed and ultrasound treated colloid was pH-neutral (7.0–7.5). When the TNs were supplied to the PGPR growth medium, dense and coarse bacterial clumps were formed which were more developed than the clumps formed in a self- produced biofilm (Supplementary FS2). The aggregates of TNs were clearly visible on the surface of bacteria in both SEM and AFM images (where the characteristic sizes of 50–60 nm could clearly be traced, (Fig. 2). The area EDS analysis of the biofilms was showing 7.5% of Ti in average among the heavier elements, while the spot analysis on nanoparticle aggregates revealed up to70–85% of Ti, showing that the particles were present in undigested form.

Single and double inoculations were performed in two model systems, in peat soil and sand, both with and without TNs (Figs 4 and 5). The peat soil represents the model system that lacks Si nanoparticles (Fig. 3). Sand is rich in natural silicates, which function similarly to TNs (Fig. 3). Earlier we have shown that AZP2 inoculation significantly increased plant drought tolerance^33^. Along with AZP2 drought tolerance enhancement, we show that harsh environment strains *P*. *polymyxa* A26 and *A*. *faecalis* AF induce plant drought stress tolerance. All three bacterial isolates (AZP2, A26 and AF) are capable of enhancing salt stress tolerance, and antagonizing the *F*. *culmorum* pathogen in the two model systems (Figs 4 and 5). TN formulation did not improve the PGPR performance in the case of single inoculations (Fig. 4A). Fascinatingly, major differences in seedlings biomass were observed when double inoculations with TNs were performed in peat soil. AZP2TN and A26TN as well as AZP2TN and AFTN combinations resulted in significantly enhanced colonization about a 25% plant biomass increase (Figs 4B, 6, 7, 8 Tables S1 and S2). Regression analysis indicates that there is a positive interaction between TN-formulated second PGPRs and seedling biomass and plant biomass increases with increased colonization of the second inoculant in all stress situations (Figs 7, 8, 9 and Table S3). This indicates that there are interactions with seedling biomass accumulation and it is crucial that both PGPR populations are associated with the roots. The results reveal the importance of PGPR root attachment, which are facilitated by TN formulation (Figs 2 and 8). Root colonization creates the base of the PGPR activity. Growth-promoting compounds produced by the bacteria need to be released in the vicinity of the plant roots in the zone of rapid uptake. We speculate that the TN formulated PGPR form micro-niches around the roots which are entirely different from the surrounding microbiome and allow the beneficial bacteria to work as a functional unit. The PGPR enhanced attachment does not fully explain the observed effect as increased colonization under normal conditions didn’t result in significant growth promotion (Fig. 4B and Table S2). It is clear that stress triggers the growth promoting effect of the harsh environmental isolates. Mechanisms of the observed effect are likely to be related to nutritional and bioactive compound release characteristic to the bacteria which coevolved with plant roots over millennia. The bacteria have superior potential to enhance plant stress tolerance^4,33^. The dynamics of the interactions are yet to be elucidated and modelled in the system established here.

Compared to peat soil, the TN-formulated strains performance was different in sand. The TN-treated PGPR inoculations did not result in significant plant biomass improvement compared to PGPR treatment without TNs (Fig. 5). The most abundant class of minerals in all soils is silicates, and various silicate particles, with a similar function to TNs, are common in most soils, especially in sands (Fig. 3). This is due to constant physical /chemical weathering and rearrangement of constituents by high biological activity. Nanosilica beneficial effects on soil bacteria and plants are well reported^25,39,40^. The silicate particles, in the same way as TNs, facilitate soil-mulch biofilm formation on plant roots. The matrix assisted by natural silicate particles protects the PGPR cells and attaches them successfully to the place of action. The results are further supported by our former studies showing that bacteria and plants can facilitate the production of natural nanomaterials that enhance aggregation, functioning as a glue between bacteria and roots^30,33^.

Multiple factors influence the PGPR effect under natural conditions. Firstly, soil as an environmental matrix is rich in various nanoparticles that have a substantial impact on the soil characteristics. Soil is also an omnipresent habitat where microbes network with their biotic components and among themselves. Hence the effectiveness of the PGPR is related to the effectiveness of the technology used for PGPR formulation. Are the cells protected from the unfavorable environment and secondary effects that hinder their performance? We show that TN formulation establishes significantly larger and thicker bacterial clumps than those observed in the case of the self-produced biofilm matrix without TNs (Fig. 2). This is in agreement with earlier observations when *B*. *amyloliquefaciens* was applied to canola roots^30^. Based on the results, we conclude that the method of inoculum delivery using TNs is applicable in soil systems and is especially efficient when several inoculants are used. Since most soils are regarded as being oligotrophic, natural nanosilica- based stimulation may trigger beneficial effects of PGPRs and can improve crop yield^39^. However, with sand as a growth substrate, the problem with nutritional deprivation will occur, as sands are poor in nutritional elements (Fig. 3). Likewise, it is hard to predict the efficiency of heterogeneous particles of varying nature, size and amount in sand soils. Still, the presence of naturally existing nanoparticles must be considered when applying TNs.

Here we studied the harsh environment PGPR strains separately and in combination with one another for the ability and potential to enhance drought and salt tolerance and to suppress the pathogen *F*. *culmorum*. The kind of investigations are challenging due to their complexity and effect collinearity. Hence to reduce the number of variables we established a model system and the results presented illustrate the potential of harsh environment isolates which may not manifest fully under more natural settings. Different treatments reveal a common pattern showing that AZP2 and A26∆*sfp* perform best under drought, AF under salt and A26 under *F*. *culmorum* pathogen stress (Figs 4 and 5). Earlier, we have shown that the biofilm and its components are part of the mechanism of AZP2 performance^33^. The action and mechanisms of each harsh environment isolate are predicted to vary and remains to be elucidated separately for every condition^18^.

Mutation of A26 by inactivating (i.e., knocking out) the *sfp* gene dramatically improved drought stress adaptation of wheat primed by A26Δ*sfp* as compared to wheat primed by the A26 wild type^9^. This *sfp* gene inactivation resulted in about the same dry weight improving characteristics as AZP2. A26∆*sfp* improved plant dry weight by 27% under no stress conditions (Fig. 5A). Under drought stress A26∆*sfp*, similarly to AZP2, improved plant dry weight more than five times. The results here support our previous results on A26∆*sfp* drought stress tolerance enhancement^9^. In addition to enhancing drought tolerance, the mutant enhanced wheat seedlings’ antagonism to the pathogen *F*. *culmorum* and salt tolerance in a rate comparable to the best performers, A26 and AF, respectively (Fig. 5A). Hence the *P*. *polymyxa* mutant with the *sfp* gene deletion has a great potential for agricultural application where transgenic cultivars are allowed. Owing to the general restraints with mutant field application in Europe, the A26∆*sfp* analysis in peat soil was not performed in the context of this study. Salt and drought are osmotic stresses similar to other osmotic stresses, e.g. those caused by heavy environmental pollution. Hence, the harsh environment isolates in combination with TNs have potential application in phytoremediation to restore marginal lands.

## Conclusions

The obtained results demonstrate that the Sol-Gel synthesized TNs stably attach PGPRs to plant roots and can be efficiently used for PGPR formulation in the case of double inoculation. We suggest that TNs aid PGPR root attachment and bacterial performance under the conditions in which diverse factors affect the plant simultaneously. The bacterial layers can be used as a tool for rational design of holobionts in food security programs. Our studies illustrate the importance of considering natural soil nanoparticles for PGPR application and may explain the generally observed inconsistent behavior of PGPRs in the field.

The TN effects are thought to be related to the particles’ ability to modulate the surface formation of complexes with important types of bio-molecules such as phospholipids and proteins^31^. Understanding the pathways would improve the effectiveness of TNs and optimize the mutually complex interactions, as it would allow their regulated delivery and release, focusing on specific tissues and cells at specific times. This would ensure that the great potential of PGPR science would find its way to facilitate reproducible field application and sustainable food production in a changing climate. The systems developed in this study serve for quantitative modelling of these interactions.

## Material and Methods

### Experimental setup

The experiments consisted of randomized block design of winter wheat (*Triticum aestivum* cv. Stava) PGPR treatments in plastic pots filled with 450 g growth substrate. Two substrates of contrasting Si nanoparticle abundance, peat soil (Sol mull, Hasselfors), or sand (Silver Sand, Sibelco) were used. Peat soil contains traces of various elements but not Si, while sand is rich in Si nanoparticles. Wheat seedlings were inoculated with PGPRs and their combinations with and without TNs as described under ‘Plant treatment’ and Table 2. Following 10 days of seed germination, three stress treatments, drought, salt and pathogen stress, were applied for 14 days. Seedlings were harvested, and TN treatment effect on shoot biomass and bacterial root colonization was estimated. Data were subjected to statistical analysis as described in ‘Data confirmation and validation’.

**Table 2:** Summary of experimental design.

**Time (days)**	**1**	**5**	**8**	**10**	**24**
Seed sterilization	Ethanol, NaOCl and sterile water				
Single PGPR strain^1^ inoculation^2^		AZP2; A26; AF; A26∆sfp			
Single titania-formulated PGPR inoculation		AZP2TN; A26TN; AFTN; A26∆sfpTN			
Double PGPR inoculation		AZP2	A26 or AF		
Double titania-formulated PGPR inoculation		AZP2TN	A26TN or AFTN		
Stress treatment				Drought; 250 mM NaCl; *Fusarium culmorumUK*	
Harvest					Plant and bacterial biomass analysis

Randomized block design was applied to winter wheat (*Triticum aestivum* cv. Stava) PGPR treatments in two growth substrates: peat (Sol Mull, Hasselfors), and sand (Silver Sand, Sibelco). Data were subjected to statistical analysis as described in ‘Data confirmation and validation’. ^1^see Table 1; ^2^see Material and Methods.

### Synthesis and characterization of nanoparticles

Uniform sized TNs were produced by acidic hydrolysis of titanium ethoxide, Ti(OC_2_H_5_)_4_, modified with triethanolamine, N(C_2_H_4_OH)_3_. For stabilization of the initial colloid solution a modification of the approach originally reported by us was used^31^. Briefly, 8 ml of Ti(OC_2_H_5_)_4_ was dissolved in 12 ml of anhydrous ethanol (obtained by distillation of absolute ethanol, Solveco, over barium ethoxide) and then 1.2 ml of N(C_2_H_4_OH)_3_ dried over 4 Å molecular sieves overnight and was added to the obtained solution in a dry box. To the produced clear solution, 3 ml of a solution of 0.5 M nitric acid solution in water was added dropwise with constant stirring. The originally transparent medium slowly turned milky and precipitation of TNs occurred overnight as white sediment. The product was washed three times with 10 ml ethanol and once with 10 ml water and air-dried at room temperature.

### Microbial agents and culture conditions

*B*. *thuringiensis AZP2*^33^ and *P*. *polymyxa* A26^4^ were isolated from the ponderosa pine rhizosphere at Mt. Lemmon, USA and from South Facing Slope at the natural laboratory called The Evolution Canyon (Table 1). *P*. *polymyxa* A26 Sfp-type 4-phosphopantetheinyl transferase deletion mutant strain (A26∆sfp), was previously generated as described^9,33,34,36^. Stock cultures were stored at −80 °C and streaked for single colonies on de Man Rogosa and Sharpe (MRS) agar containing 2 M NaCl. All bacterial strains were grown in half-strength tryptic soy broth (1/2 TSB; pH 6.2) at 30 ± 2 °C for 24 hours. The fungal pathogens *Fusarium culmorum* strain UK^41^ were used to challenge plants with pathogen. The pathogens were grown on potato dextrose agar (PDA) plates at 22 °C. *Alcaligenes faecalis* AF (SEQ ID: KY563695) was isolated as a ponderosa pine endophytic isolate from ponderosa pine (*Pinus ponderosa*) roots grown on nutrient-deprived gneiss rock at Mt Lemmon, AZ, USA (N 32° 23.1408’ W 110° 41.6315’) at an elevation of 2150 m by methods used by us earlier^33^. Briefly, the plant roots were shaken and washed in sterile distilled water to remove all loosely attached soil and rock powder and to collect bacteria intimately linked to the plant root. Plants were placed in sterile plastic bags, transferred to the laboratory, and then stored at + 4 °C until they were processed the next day. The roots were excised and subjected to surface sterilization procedure: 60 s wash in 99% ethanol, followed by a 6 min wash in 3% NaOCl, a 30 s wash in 99% ethanol, and a rinse in sterile water. The surface sterilized root material (1 g) was homogenized as described by the manufacturer using sterile vessels and FastPrep Instrument (BIO 101® Systems). The homogenised material was suspended in sterile PBS (137 mM NaCl, 2.7 mM KCl, 10 mM Na_2_HPO_4_, 2 mM KH_2_PO_4_, pH 7.4). The rhizosphere suspension was then salt- treated with TSB containing 2 M NaCl for 24 h. Tryptic Soy Agar (TSA) plates containing 2 M NaCl were inoculated with 100 µL of these suspensions, corresponding to 10^−3^–10^−5^ g plant rhizosphere material per plate. All agar media contained 15 g agar and 50 mg cycloheximide, to reduce fungal growth, and had a pH of 7. The inoculated petri dishes were incubated for several weeks at 30 °C in boxes together with a beaker of water (to prevent drying of the agar). The bacterial colonies were studied for plant drought and salt stress tolerance enhancement biotests. Water stress was studied by 10 day inoculated seedlings water withdrawal and salt stress by watering the 10 day old seedlings with 250 mM NaCl solution. The efficient strains were identified by 16S rDNA sequencing. Aliquots of 10 mM of primers 1492 R (5′-GGTTACCTTGTTACGACTT-3′) and 27 F (5′-AGAGTTTGATCCTGGCTCAG-3′) and 1 µl of template were used. The reaction was performed in 10 µl. The reaction conditions were 95 °C for 2 min followed by 30 cycles of denaturation at 95 °C for 15 s, annealing at 55 °C for 20 s, primer extension at 72 °C for 1 min, followed by the final extension at 72 °C for 5 min. For sequencing, the PCR products were purified with the QIAquickTM Gel Extraction kit (QIAGEN, Hilden, Germany). Screening for bacterial metabolic properties and plant drought tolerance enhancement was performed as described earlier^4^.

### Bacterial growth in the presence of nanoparticles

Strains AZP2, A26, AF were grown in 1/2 TSB with 50 µg ml^−1^ TNs at 30 ± 2 °C for 24 hours. TN’s were deagglomerated 1–5 min by ultrasound and mixed with growth medium prior bacterial inoculation. Culture density was determined by colony forming unit analysis (CFU). For the mock treatment 1/2 TSB with 50 µg ml^−1^ TNs was used.

### Plant treatment

Wheat seeds were surface sterilized by 60 s wash in 99% ethanol, followed by a 6 min wash in 3% NaOCl, a 30 s wash in 99% ethanol, and a rinse in sterile water. Single inoculations were performed by watering the 5 day seedlings with 1 ml of strains AZP2, A26, AF and/or A26∆*sfp* ten times deionized water diluted samples (10^5^ cells per ml) (Table 2). Bacteria were grown in 1/2 TSB at 30 ± 2 °C for 24 hours (see Microbial agents and culture conditions). For TN treatments, root inoculations were performed with 1 ml of AZP2TN, A26TN and AFTN 10 times sterile water diluted samples. Double inoculations AZP2/A26, AZP2/AF and AZP2TN/A26TN, AZP2TN/AFTN were performed sequentially. Five day old seedlings were inoculated with AZP2 or AZP2TN cells as described above and in 3-day intervals respectively A26, AF or A26TN, AFTN inoculations were performed (Table 2). In addition to root treatments, shoot treatments were performed by spraying 5 day old seedlings with one ml of corresponding 24 h culture filtrates (without TNs). In case of double inoculation, all seedlings were sprayed with AZP2 culture filtrates (without TNs). For the mock treatment 1/2 TSB was used.

Both inoculated and non-inoculated treatments were replicated five times. Double inoculations with larger variation treatments were replicated six times. The pots, three plantlets per pot, were incubated in a controlled environment in a MLR-351H (Phanasonic, IL, USA) growth chamber with 24/16 °C (day/night) temperature, and 16 h photoperiod at a quantum flux density of 250 μmol m^−2^ s^−1^. The soil moisture was adjusted to 75% of soil water-holding capacity (12.5% of soil dry mass) using deionized water. No nutrition was provided but soil moisture content was kept constant during the first 10 days of seedling growth. Following 10 days of seed germination, drought stress was induced by stopping watering. Plants were drought-stressed for 14 days. Soil volumetric water content was measured using 5TE soil moisture sensors (Decagon Devices, Inc., Pullman, WA, USA). For salt stress 10-day-old plantlets were exposed to long stress by watering with 250 mM NaCl solution. Pathogen stress was induced for 14 days by inoculating 10-day-old plants with *F*. *culmorum* pathogen by incubation with an agar slice of 1-week-old culture^41^.

### Plant and bacterial biomass analysis

Plant biomass is expressed as shoot dry mass recorded after 14 days drought, salt or pathogen stress exposure in sand and peat soil. Wheat shoot samples were dried at 105 °C to a constant mass, cooled and weighed. Bacterial biomass (C-content) was calculated based of bacterial quantification (see Supporting Material) as described by Bratback, 1985^42^ and was used as a proxy for root colonization plant biomass regression analysis.

### Scanning electron microscopy

Environmental scanning electron microscopy (ESEM) micrographs of the samples were obtained with a Hitachi TM-1000-μDex variable pressure scanning electron microscope. Samples were deposited on a carbon tape and coated with gold using Sputter Coater 108 auto (Cressington) when the samples included biofilm. TNs were investigated in the form of dry xerogel.

### Transmission electron microscopy

Transmission electron microscopy (TEM) images of TN samples were obtained using a Topcon EN-002 B ultrahigh resolution analytical electron microscope. Data are courtesy of the EM Research Center of the Claude Bernard Lyon-1 University.

### Atomic Force Microscopy

Bruker FastScan Bio Atomic Force Microscopy (AFM) operating in tapping mode was used for surface structure characterization.

### Data confirmation and validation

To ensure reproducibility, three biological replicates of every single PGPR treatment, which showed relatively small variation, were performed. In the case of double inoculations five biological replicates were performed. Replicated data were studied for normal distribution and analysed by MiniTab17 ANOVA. TN treatment effects were considered statistically significant, *p* ≤ 0.01 or nonsignificant (ns). Univariate analysis of selected samples involving distribution and variability of distribution was performed using the Unscrambler X10.4.1 descriptive statistics (box plot). Linear regressions (Unscrambler X10.4.1) were used to determine the relationships between seedling biomass accumulation and root colonization.

## Electronic supplementary material

Supporting Information
