# Supplementary material for: Titania (TiO2) nanoparticles enhance the performance of growth-promoting rhizobacteria
Source: Sci Rep. 2018 Jan 12;8:617. doi: 10.1038/s41598-017-18939-x (PMC5766586; doi:10.1038/s41598-017-18939-x)
Supplement: Supplementary file 1 — Supporting Information [file 41598_2017_18939_MOESM1_ESM.pdf]

Supporting Information

Titania (TiO_2) nanoparticles enhance the performance of growth-promoting rhizobacteria

Salme Timmusk[1,3*], Gulaim Seisenbaeva[2], and Lawrence Behers[1,3]

AZP2, A26 and AF identification and quantification

Plants were randomly selected to confirm that the bacterial biofilm on the surface and inside the roots consisted of strains AZP2, A26 or AF after 24 days of growth [1] . Briefly, the roots of selected plants were shaken and washed in sterile distilled water to remove loosely attached soil and to collect bacteria intimately linked to the plant root. Thereafter, the roots were homogenized and the content of endospore-forming and heat resistant bacteria was determined after heat treatment of the soil or plant material suspension at 80ºC for 30 min. TSA 2M NaCl plates were inoculated with 100 ml of these suspensions, corresponding to 10^{-3}–10^{-5}g soil or plant rhizosphere material per plate. Colonies characteristic for AZP2, A26 and AF were counted. For identity conformation, DNA was isolated from 1-day-old cultures on agar plates. Single colonies were re-suspended to obtain a bacterial density of about 10^5 colony-forming units per ml of suspension. A 0.3 ml aliquot of the bacterial culture was suspended in 4.7 ml of buffer (10 mM Tris-HCl, pH=7.6, 50 mM KCl, 0.1% Tween 20). For lysing, the suspension was heated to 95ºC and immediately cooled on ice. The mixture was centrifuged at 6000 x *g* for 5 min and the supernatant was used for PCR analysis. Aliquots of 10 mM of primers 1492R (5'-GGTTACCTTGTTACGACTT-3') and 27F (5'-AGAGTTTGATCCTGGCTCAG-3') and 1 µl of template were used. The reaction was performed in 10 µl. The reaction conditions were 95ºC for 2 min followed by 30 cycles of denaturation at 95ºC for 15 s, annealing at 55ºC for 20 s, primer extension at 72ºC for 1 min, followed by the final extension at 72ºC for 5 min. For sequencing, the PCR products were purified with QIAquickTM Gel Extraction kit (QIAGEN, Hilden, Germany).

Bacterial biomass (C-content) calculated as described by Bratbak, 1985[2] was used as a proxy for root colonization plant biomass regression analysis.

Supplementary Table 1

Table S1 Analysis of variance showing effects of TN treatment on plant biomass accumulation.

Significant effects are shown in bold (P(F)<0.001)

**PGPR Stress**	**TN treatment**	
	**F -ratio**	**P(F)**
**Control Drought**	1.02	0.18
**AZP2/A26**	**12.19**	**0.003**
**AZP2/AF**	**20.2**	**0.001**
**Control Salt**	0.2	0.9
**AZP2/A26**	**21**	**0.001**
**AZP2/AF**	**210**	**<0.001**
**Control Pathogen**	0.1	0.7
**AZP2/A26**	**15.5**	**0.001**
**AZP2/AF**	**15.4**	**0.001**

Supplementary Table 2

Table S2 Analysis of variance showing effects of TN treatment on PGPR colonization

Significant effects are shown in bold (P(F)<0.001)

**PGPR Stress**	**TN treatment**	
	**F -ratio**	**P(F)**
**Drought**		
**AZP2**	0.2	0.66
**A26**	**326**	**<0.001**
**AF**	**290**	**<0.001**
**Salt**		
**AZP2**	0.12	0.46
**A26**	**310**	**<0.001**
**AF**	**290**	**<0.001**
**Pathogen**		
**AZP2**	0.4	0.35
**A26**	**299**	**<0.001**
**AF**	**281**	**<0.001**
**Unstressed**		
**AZP2**	0.2	0.66
**A26**	**290**	**<0.001**
**AF**	**292**	**<0.001**

Supplementary Table 3

Table S3 Bacterial (second inoculant) colonization correlation with plant biomass accumulation. Significant effects are shown in bold (P(F)<0.05)

**PGPR Stress**	**TN treatment**	
	**R**	**P(R)**
**Drought AZP2**	0.02	0.96
**Drought A26**	**0.59**	**0.006**
**Drought AF**	**0.61**	**0.035**
**Salt AZP2**	0.011	0.9
**Salt A26**	**0.54**	**0.014**
**Salt AF**	**0.89**	**<0.01**
**Pathogen AZP2**	0.01	0.8
**Pathogen A26**	**0.473**	**0.035**
**Pathogen AF**	**0.462**	**0.04**

Supplementary figures

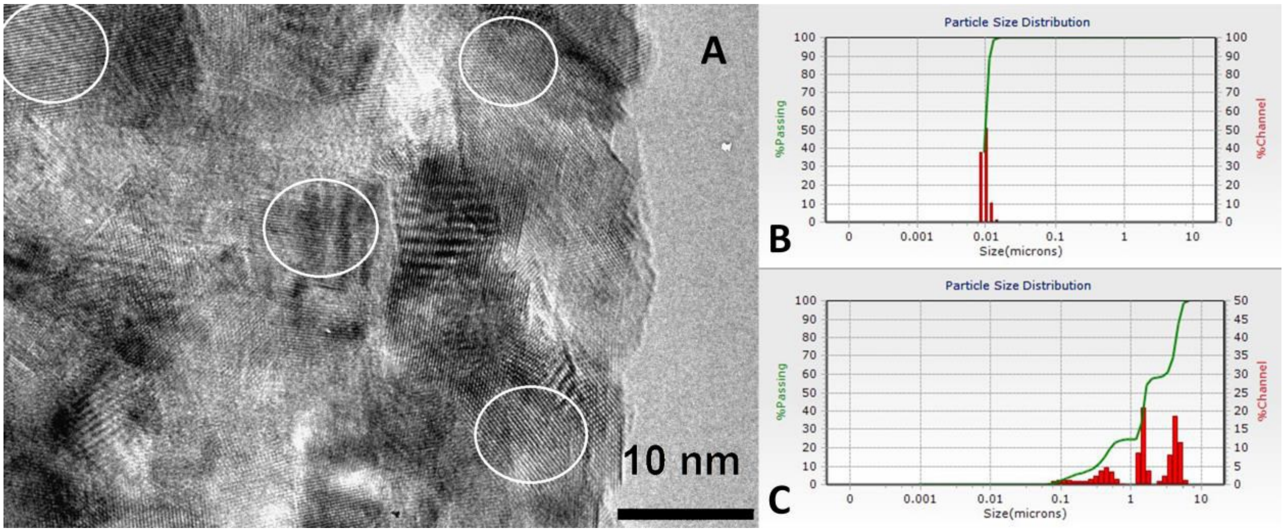

Figure S1

TEM image of the dried dispersion of the applied TiO_2 nanoparticles produced using modified methodology from ref. 18 (A). Hydrodynamic size of the particles in water (B) and in isotonic salt solution (C, 0.5 ml of dispersion diluted by 10 ml isotonic NaCl), both by DLS as reported in [3].

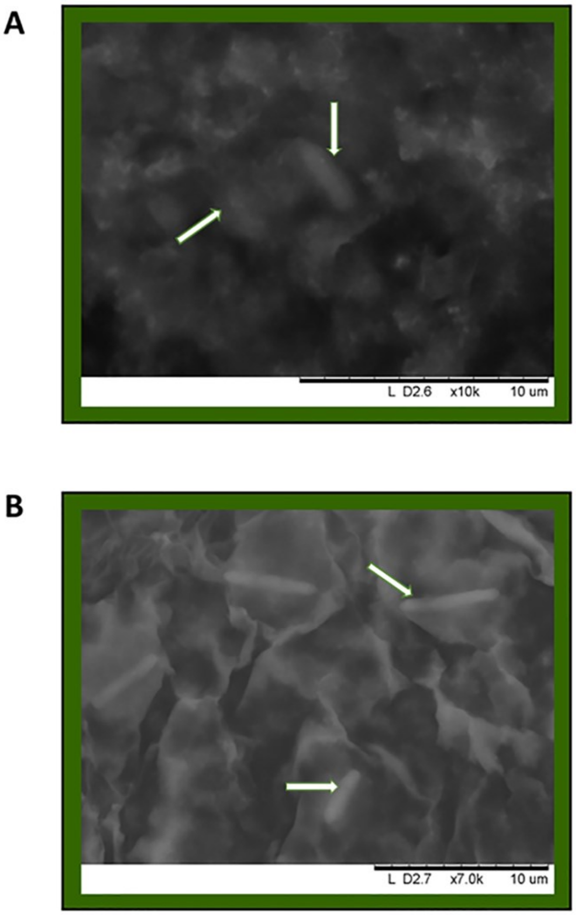

Figure S2
ESEM micrographs of *Bacillus thuringiensis* AZP2 cells (A) and cells grown with TNs for 24 hours (B). Note AZP2TN dense agglomerates thicker than observed in case of AZP2 biofilm. Arrows indicate bacterial cells.

References:

1

1 Timmusk, S. *et al.* Drought-tolerance of wheat improved by rhizosphere bacteria from harsh environments: enhanced biomass production and reduced emissions of stress volatiles. *PloS ONE* 1-13 doi:10.1371/journal.pone.0096086 (2014).

2 Bratbak, G. Bacterial biovolume and biomass estimations. *Applied and Environmental Microbiology* **49**, 1488-1495 (1985).

3 Seisenbaeva, G. A. Dispersion of TiO2 nanoparticles improves burn wound healing and tissue regeneration through specific interaction with blood serum proteins. *Sci. Rep. DOI: 10.1038/s41598-017-15792-w* (2017).

2
